# Supplementary material for: Amphiphilic Particle-Stabilized Nanoliter Droplet Reactors with a Multimodal Portable Reader for Distributive Biomarker Quantification
Source: ACS Nano. 2023 Oct 12;17(20):19952–60. doi: 10.1021/acsnano.3c04994 (PMC10604076; doi:10.1021/acsnano.3c04994)
Supplement: Supplementary file 1 — nn3c04994_si_001.pdf [file nn3c04994_si_001.pdf]

Supporting Information

# Amphiphilic particle-stabilized nanoliter droplet reactors with a multi-modal portable reader for distributive biomarker quantification

Vishwesh Shah*[1], Xilin Yang*[2], Alyssa Arnheim[1], Shreya Udani[1], Derek Tseng[2], Yi Luo[2], Mengxing Ouyang[1], Ghulam Destgeer[7], Omai B. Garner[4], Hatice C. Koydemir[5,6], Aydogan Ozcan[†,2,3], Dino Di Carlo[‡,1,3]

1. Department of Bioengineering, University of California - Los Angeles, Los Angeles, California 90095, United States

2. Department of Electrical and Computer Engineering, University of California - Los Angeles, Los Angeles, California 90095, United States

3. California Nanosystems Institute (CNSI), University of California - Los Angeles, Los Angeles, California 90095, United States

4. Department of Pathology and Laboratory Medicine, University of California - Los Angels, Los Angeles, California 90095, United States

5. Center for Remote Health Technologies and Systems, Texas A&M Engineering Experiment Station, College Station, Texas 77843, United States

6. Department of Biomedical Engineering, Texas A&M University, College Station, Texas 77843, United States

7. Department of Electrical Engineering, Technical University of Munich, Munich 80333, Germany

*contributed equally

‡Corresponding author: dicarlo@ucla.edu

†Co-corresponding author: ozcan@ucla.edu

# Table of Contents

Supplementary Table 1: Cost Breakdown of the Portable Image Reader

Supplementary Figure 1: Improved 3D Printed Co-Axial Flow Fabrication Device

Supplementary Figure 2: Particle Recognition on the Multimodal Reader

Supplementary Figure 3: Regional Intensity and ROI

Supplementary Figure 4: rNT-ProBNP Spiked in Plasma-EDTA

Supplementary Figure 5: Reduction in Measurement Error Due to Multiple Measurements per Sample

Supplementary Figure 6: Comparison Between “Fraction Above Threshold” and “Mean Intensity” Methods

Supplementary Figure 7: Patient Sample Validation

Supplementary Table 1: Cost Breakdown of the Portable Image Reader

| Parts | Price ($) |
|---|---|
| Raspberry Pi | 75 |
| Raspberry Pi Camera V2 | 30 |
| White LED board + Side LED | ~3 |
| Green laser diodes | ~40 |
| External lens | ~10 |
| Fluorescent filter | 240 |
| Z-positioner | 492* |
| Customized 3D printed holder | ~75** |
| Total | 965 |

* We employed a high-precision manual linear stage, which is not strictly necessary. An alternative manual stage with reduced precision can be sourced for approximately $200.

** Estimate derived from the weight of the print and printing material cost.

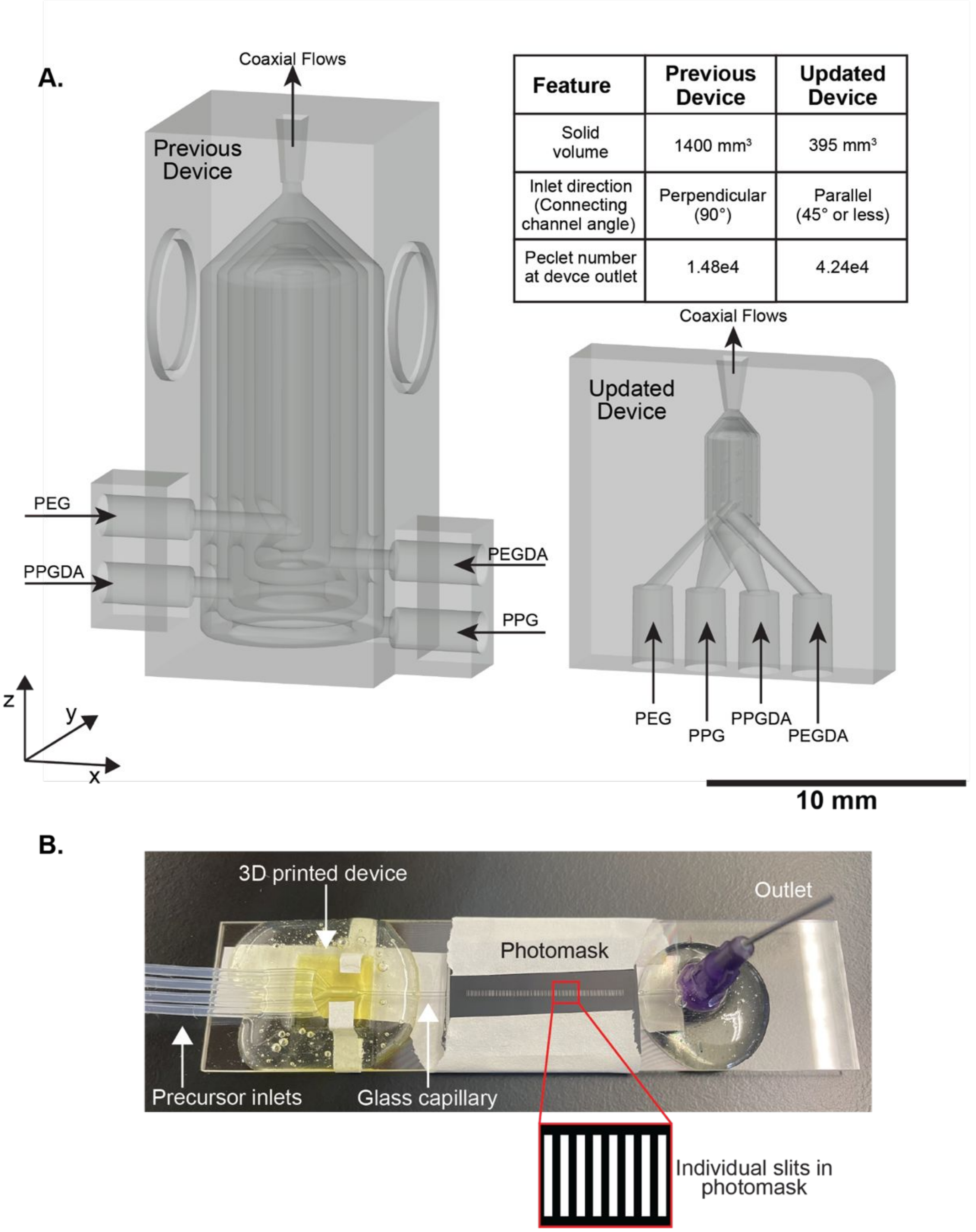


| Feature | Previous Device | Updated Device |
|---|---|---|
| Solid volume | 1400 mm³ | 395 mm³ |
| Inlet direction (Connecting channel angle) | Perpendicular (90°) | Parallel (45° or less) |
| Peclet number at devce outlet | 1.48e4 | 4.24e4 |

**Supplementary Figure 1: Improved 3D printed co-axial flow fabrication device. (A)** Comparison of previously published device (left) and improved, scaled down device (right) designs as CAD images. The new design is 3.5x smaller by volume and features inlets aligned with the direction of flow for more streamlined device assembly and fabrication. Peclet number is calculated at the point where outer two streams meet near the device outlet. **(B)** Image of an assembled device where the 3D printed device is connected to inlet tubing and polymerization capillary at the device outlet. A photomask is applied over the capillary, through which UV light can be applied.

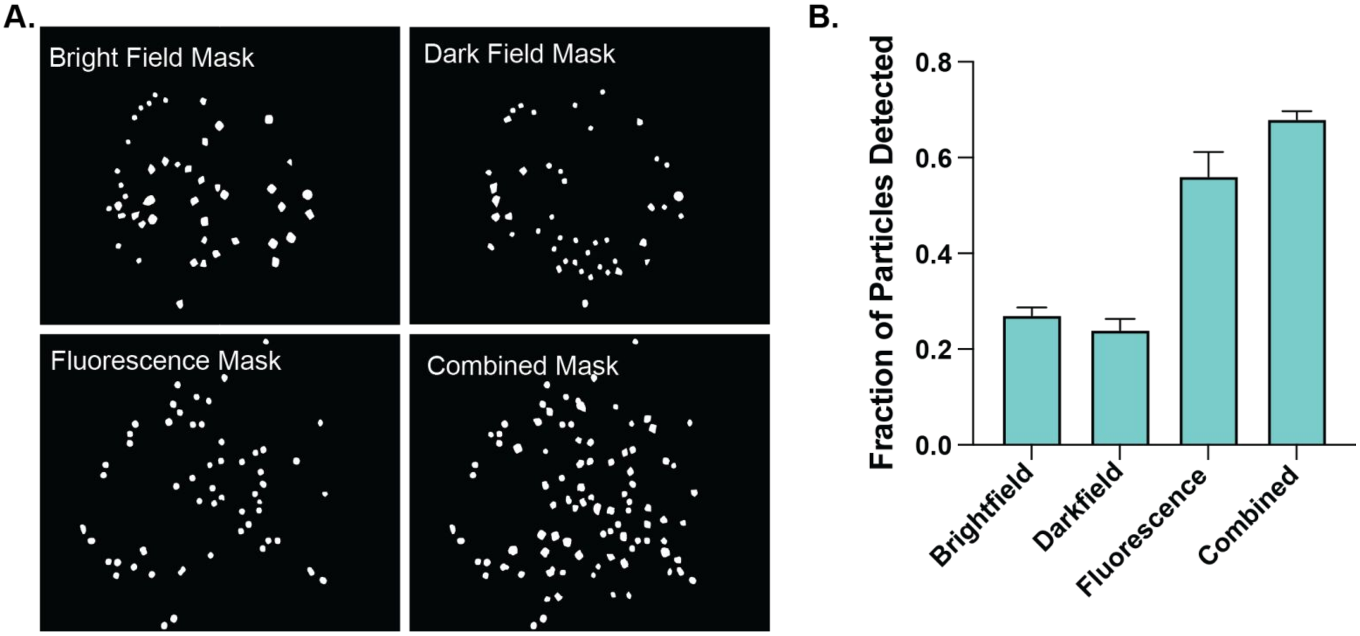


**Supplementary Figure 2: Particle Recognition on the Multimodal Reader. (A)** Images showing particle masks identified from individual brightfield, darkfield, and HDR fluorescence images, and a combined (fused) mask. **(B)** Combining particle masks from multiple imaging modalities enables detection of a larger fraction of particles per well. Data shown is from 3 repeats and error bars represent standard deviation.

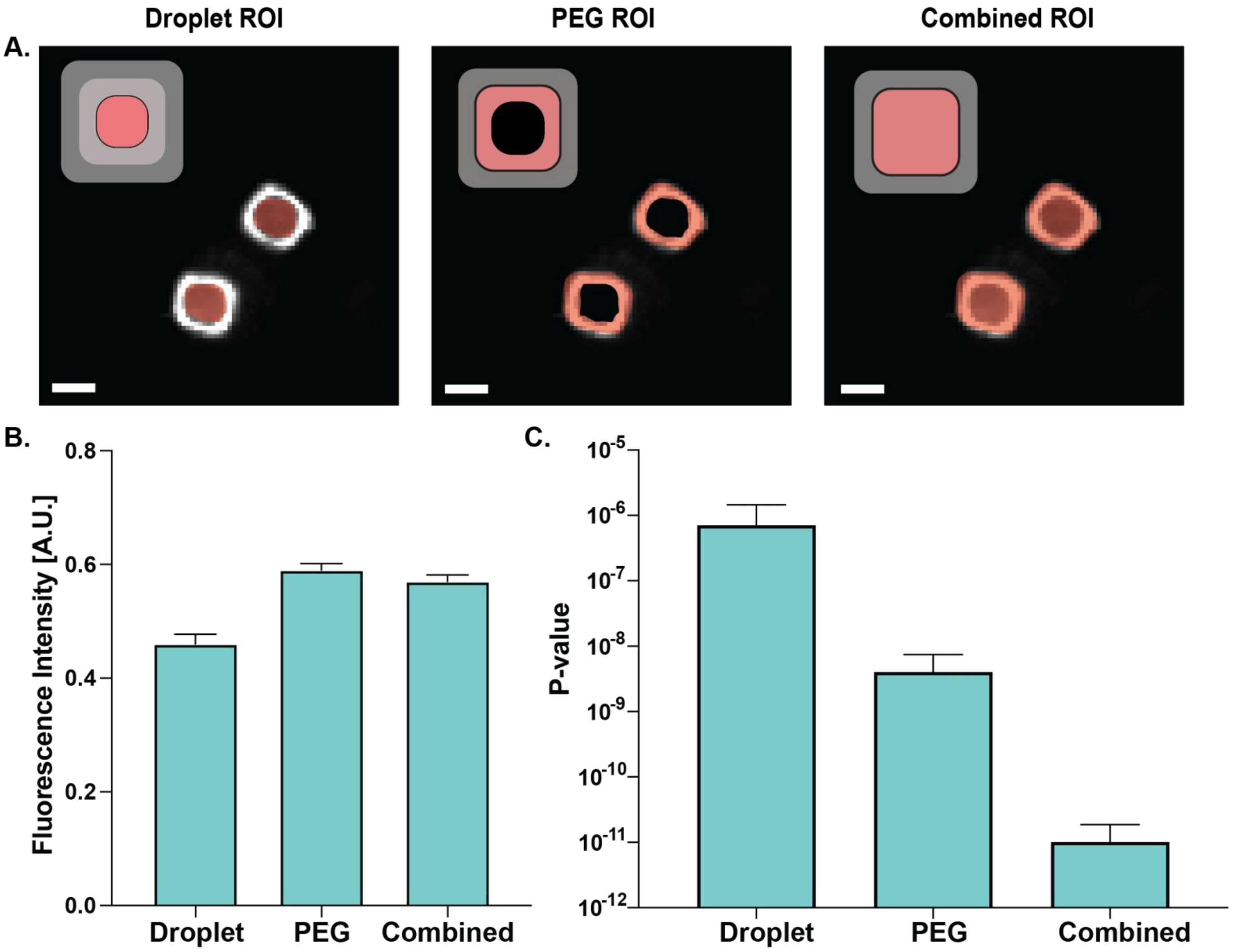


**Supplementary Figure 3: Regional Intensity and ROI. (A)** Three different types of ROI enabled by the lab on a particle assay are schematically shown. The droplet ROI encompasses only the internal droplet in the particle cavity, the PEG ROI consist of only the inner PEG region of the amphiphilic PPG/PEG particles, and the combined ROI includes both the droplet and PEG region in the fluorescence intensity measurements. **(B)** Mean intensity calculated from the three different ROIs for n = 82 particles at an NT-proBNP spike concentration of 0.1 ng/ml. Error bars show standard deviation. **(C)** Statistical significance between intensity distributions of negative control (0 ng/ml) and clinical cut-off (0.1 ng/ml) for each of the ROIs. Data shown is from 3 individual repeats, each repeat includes >50 individual particles detected. Error bars show standard deviation between repeats. The combined ROI enables the highest statistical significance in differentiating across the cut-off.

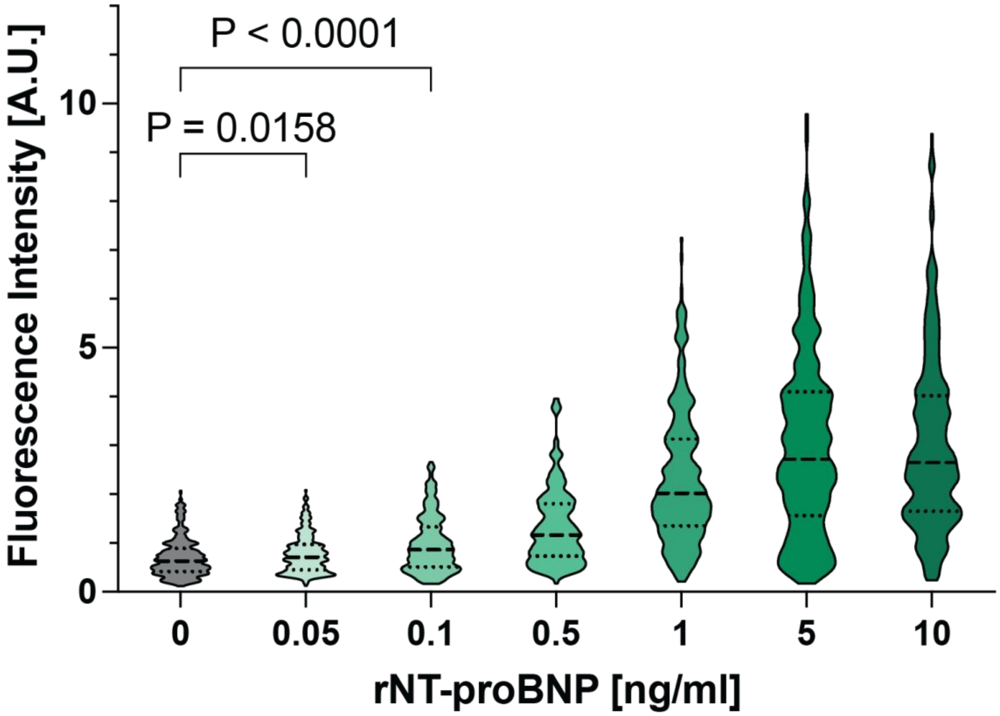


**Supplementary Figure 4: rNT-proBNP spiked in plasma-EDTA.** Violin plots showing distributions of particle intensities as a function of spiked rNT-proBNP concentration.

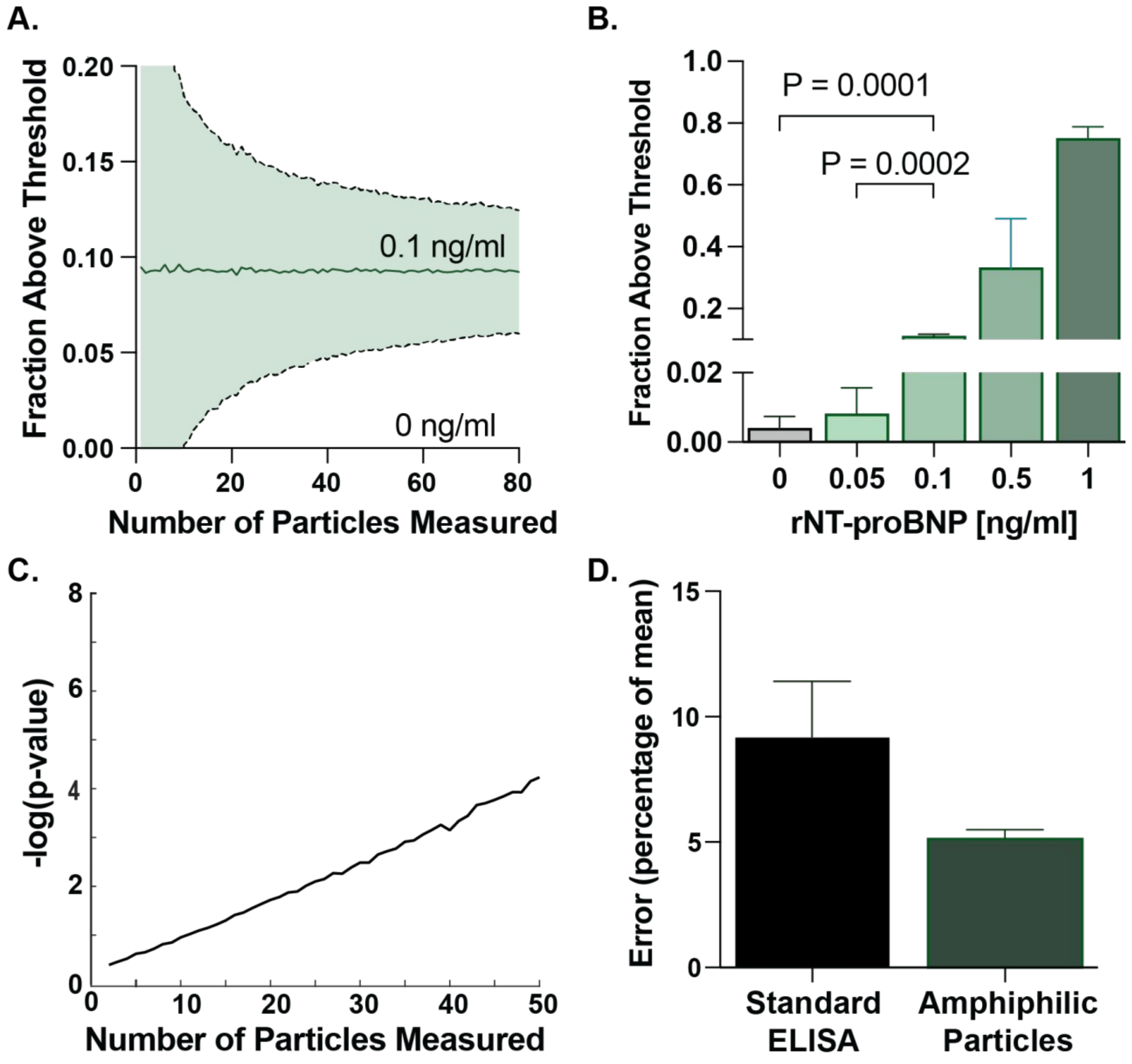


**Supplementary Figure 5: Reduction in measurement error due to multiple measurements per sample.** **(A)** Monte Carlo simulations (n = 5000) show how the variation in fraction of particles above threshold (set from distribution of negative control particle-drop intensities), changes as a function of number of particles measured. As more particles are measured, the error rate of designating a sample at clinical cut-off (~0.1 ng/ml) as a false negative exponentially decrease. **(B)** Mean fraction above threshold for varying concentrations of NT-proBNP spiked in plasma-EDTA. Error bars show standard deviation. **(C)** Statistical capability to differentiate between distributions of 0 and 0.1 ng/ml increases as a function of particles measured per sample. **(D)** Mean variation (error) in measurement, from standard well plate based ELISA and amphiphilic particle based ELISA, across 6 cardiac patient samples

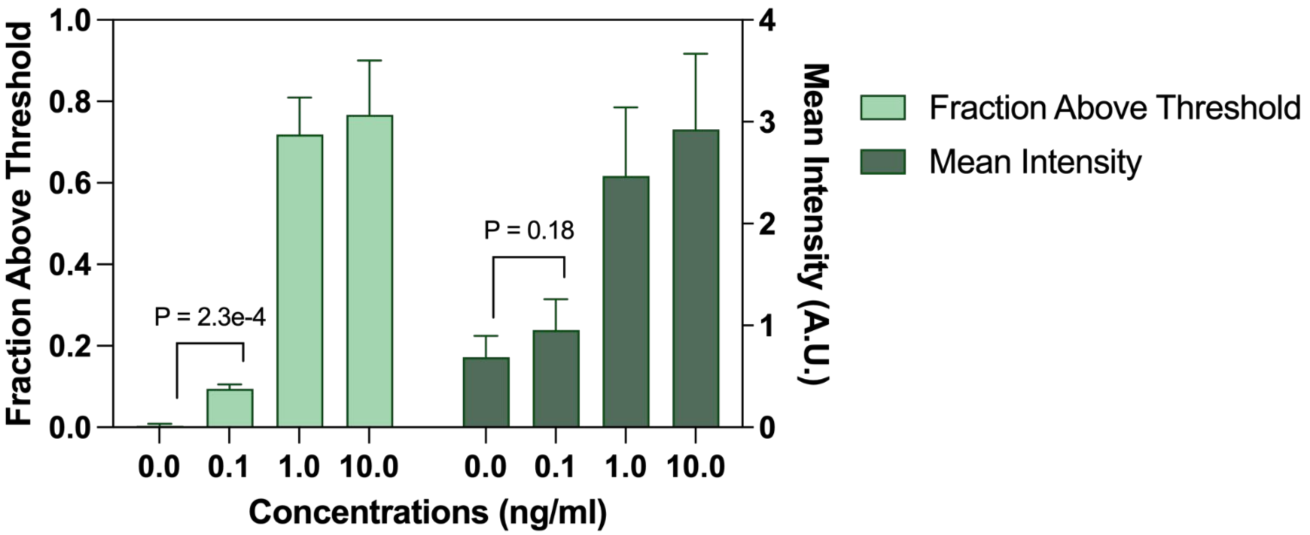


**Supplementary Figure 6: Comparison Between “Fraction Above Threshold” and “Mean Intensity” Methods.** Graphs showing the "Fraction Above Threshold" and the "Mean Intensity" values for three repeat experiments across varying NT-proBNP concentrations. The left portion of the chart shows the "Fraction Above Threshold" which is derived from the percentage of individual particles with fluorescence intensity above a threshold compared to the negative control. The right portion of the chart shows "Mean Intensity" which is the mean fluorescence intensity of all particles in a well. P-values are shown for discriminating between 0 and 0.1 ng/ml concentrations, showing that measuring each individual particle provides increased statistical confidence in the concentration difference.

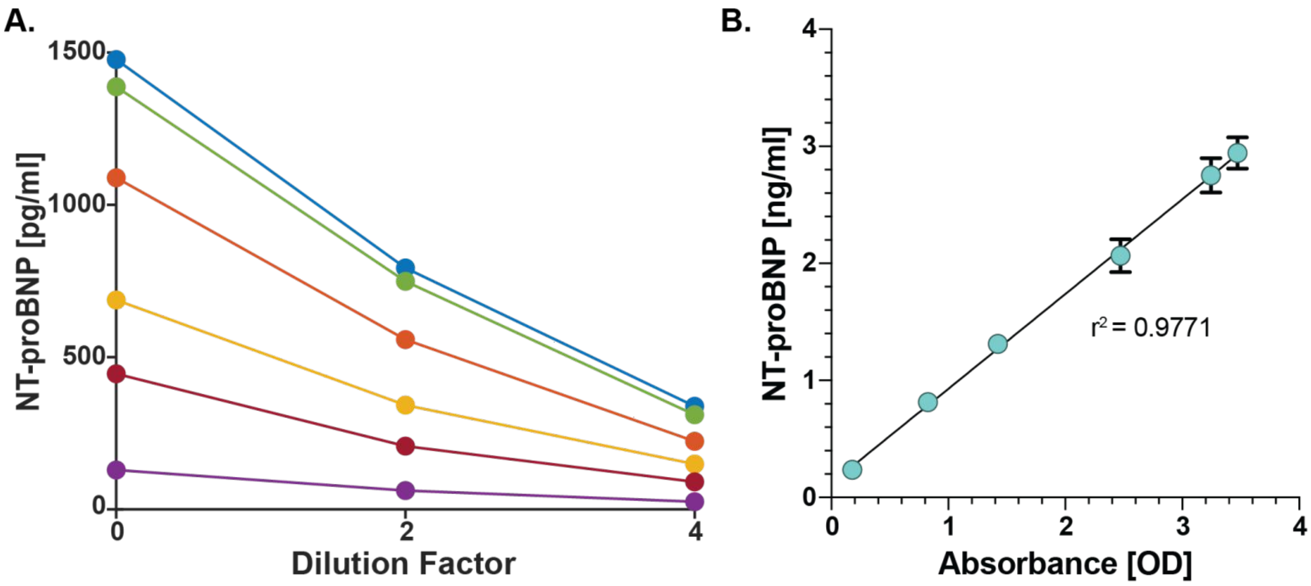


**Supplementary Figure 7: Patient Sample Validation. (A)** Dilution curves for the six patient samples with NT-proBNP levels > 100 pg/mL validated by external clinical ELISA chosen for patient testing on lab on a particle format. The two healthy samples had NT-proBNP levels below the limits of detection of the ELISA. **(B)** Mean and standard deviation of the ground truth measurements from patient samples within the linear range of the assay. These patient samples were chosen for testing on the lab on a particle platform since they had low variation in the dilution curves and produced linear response on the clinical validation test.
